# Situational awareness within objective structured clinical examination stations in undergraduate medical training - a literature search

**DOI:** 10.1186/s12909-017-1105-y

**Published:** 2017-12-21

**Authors:** Markus A. Fischer, Kieran M. Kennedy, Steven Durning, Marlies P. Schijven, Jean Ker, Paul O’Connor, Eva Doherty, Thomas J. B. Kropmans

**Affiliations:** 1 National University Ireland Galway, School of Medicine, University Road, Galway, H91TK33 Ireland; 2 Department of Internal Medicine, University of the Health Sciences, 4301 Jones Bridge Road, Bethesda, MD 20814 USA; 3 Department of Surgery, Academic Medical Center Amsterdam, Meibergdreef 9, 1105 AZ Amsterdam-Zuidoost, The Netherlands; 4 University of Dundee. Clinical Skills Centre Level 6, Ninewells Hospital & Medical School, Dundee, UK; 5 National University Galway Ireland, Discipline of General Practice, Distillery Road, Galway, H91TK33 Ireland; 6 Royal College of Surgeons in Ireland, 123 St Stephen’s Green, Dublin 2, Ireland

**Keywords:** Situational awareness, Undergraduate medical education, Objective structured clinical examination, Diagnostic reasoning, Clinical reasoning

## Abstract

**Background:**

Medical students may not be able to identify the essential elements of situational awareness (SA) necessary for clinical reasoning. Recent studies suggest that students have little insight into cognitive processing and SA in clinical scenarios. Objective Structured Clinical Examinations (OSCEs) could be used to assess certain elements of situational awareness. The purpose of this paper is to review the literature with a view to identifying whether levels of SA based on Endsley’s model can be assessed utilising OSCEs during undergraduate medical training.

**Methods:**

A systematic search was performed pertaining to SA and OSCEs, to identify studies published between January 1975 (first paper describing an OSCE) and February 2017, in peer reviewed international journals published in English. PUBMED, EMBASE, PsycINFO Ovid and SCOPUS were searched for papers that described the assessment of SA using OSCEs among undergraduate medical students. Key search terms included “objective structured clinical examination”, “objective structured clinical assessment” or “OSCE” and “non-technical skills”, “sense-making”, “clinical reasoning”, “perception”, “comprehension”, “projection”, “situation awareness”, “situational awareness” and “situation assessment”. Boolean operators (AND, OR) were used as conjunctions to narrow the search strategy, resulting in the limitation of papers relevant to the research interest. Areas of interest were elements of SA that can be assessed by these examinations.

**Results:**

The initial search of the literature retrieved 1127 publications. Upon removal of duplicates and papers relating to nursing, paramedical disciplines, pharmacy and veterinary education by title, abstract or full text, 11 articles were eligible for inclusion as related to the assessment of elements of SA in undergraduate medical students.

**Discussion:**

Review of the literature suggests that whole-task OSCEs enable the evaluation of SA associated with clinical reasoning skills. If they address the levels of SA, these OSCEs can provide supportive feedback and strengthen educational measures associated with higher diagnostic accuracy and reasoning abilities.

**Conclusion:**

Based on the findings, the early exposure of medical students to SA is recommended, utilising OSCEs to evaluate and facilitate SA in dynamic environments.

## Background

Diagnostic and treatment errors have gained increased attention over the last decades [1, 2]. It has been suggested that these errors are intensely personal and influenced by the physicians´ knowledge and cognitive abilities such as defective information processing and verification [3–5]. Clinical Reasoning (CR) as the underlying cognitive process in diagnostic and therapeutic decision making is directed by the situation and context of the patient’s condition [6]. The ability for CR necessitates recognition and incorporation of multiple individual aspects of a patient, which enables the selection of the best treatment option in any given clinical presentation [7]. The accumulation of cognitive errors within CR has been suggested as predictive for the genesis of harmful events to the patient [8]. Notwithstanding the implementation of innovative teaching and assessment methods, such as simulation-based learning [9, 10] and problem-based learning [11, 12] into medical education curricula, flawed identification of the clinical presentation and defective appropriateness of therapeutic options continue to be reported [13–15]. Situational awareness (SA) was described by Endsley in respect to aviation as "a person’s mental model of the world around them" [16]. Knowledge about a given set of actualities is central to effective decision making and ongoing assessment in dynamic systems [6, 17, 18]. The ability to integrate successive information and identify conflictive perceptions is an essential precondition for maintaining adequate SA [17, 19]. The incorporation of the surrounding circumstances, the given set of actualities and their possible impact on future outcomes have been divided into three different levels of SA: Level 1 Perception, Level 2 Comprehension and Level 3 Projection [17]. In healthcare, SA was identified as one key element of medical practice involving multiple cognitive capacities such as perception, understanding, reasoning and meta-cognition [20]. With regard to clinical practice, SA is believed to be essential for recognising and interpreting the clinical symptoms and signs of a patients´ illness, thereby enabling accurate CR [21–24]. The WHO identified inadequate SA as a primary parameter associated with deficient clinical performance [25], recommending the implementation of “human factors” training as realised in other high-risk environments in medical undergraduate education [26]. Furthermore, SA was emphasised as one of four fundamental cornerstones incorporated in patient safety education into an undergraduate medical curriculum [27].

The development of clinical expertise is separated into four different levels [28, 29]. Students, initially characterised as “unconsciously incompetent”, learn clinically from experienced doctors who apply pattern recognition in their daily practice when assessing patients [30, 31]. Novices often are cognitively overburdened by the vast amount of available information and the prioritising process in identifying essential data, resulting in an incomplete or defective perception of the situation [32]. Professional clinicians who have developed their mental models by integration of knowledge and expertise over many years, are termed “unconsciously competent” [33]. The utilisation of illness scripts and schemata enables fast non-analytical thinking (System 1) resulting in an expeditious “big picture” of the clinical presentation of the patient, which is more comprehensive and projects possible outcomes when compared with the mental models of novices [32]. If the situation is not completely understood, clinical experts are able to switch to analytical thinking (System 2) [34]. However, they are commonly unaware of elements of SA and therefore, generally cannot convey or teach this sequence of data gathering and incorporation into the reasoning process [22, 35]. As a result, observing senior tutors might not enable students to develop incremental levels from conscious incompetence towards conscious competence through perceiving the essential steps of identifying and integrating relevant information for CR [33, 36]. Furthermore, Kiesewetter et al. emphasised, that very little knowledge exists about cognitive processing by medical students which may limit instruction on the incremental steps in CR in medical education [37]. Twenty years ago, Goss highlighted the fact, that medical students enter their third year of training competent in information gathering and facilitating patient care, but with deficient diagnostic reasoning ability [18]. Upon providing either a clinical vignette format or a chief complaint format in a paper-based examination, Nendaz and colleagues compared students, residents and general internists abilities in considering differential diagnosis (SA Level 2) or selecting basic diagnostic assessments (SA Level 1) and considering treatment options (SA Level 3). Thereby they noted that students were seen to be able to demonstrate knowledge and carry out examinations, but struggled to incorporate the data into further diagnostic processes [38]. Because the utility of the data gathering process is closely linked with the process of subsequent reasoning, both should be jointly addressed and evaluated. More recently, Schuwirth argued that the outcome based assessment does not reflect CR abilities, and therefore, adequate alternative evaluation techniques of intermediate steps should be explored [39]. Singh et al. suggested a change in the current framework of the analytical diagnostic process in order to identify breakdowns in SA. By distinguishing the level at which SA was lacking, distinct measures can be applied in subsequent training [40]. This suggests the necessity of emphasising the understanding of SA in the medical context and of formulating novel potentials to teach and evaluate the utilisation of SA in educational healthcare settings.

Objective structured clinical examinations (OSCEs) are, in theory, intended to function as an educational measure during medical training allowing for the assessment of student’s competence under variable circumstances [41, 42]. Fida and Kassab showed that scores achieved by medical students in OSCE stations demonstrated strong predictive value for the students´ ability to identify and integrate relevant information and competently manage a patient [7]. Therefore, there is potential for the identification and remediation of deficits in selecting and integrating essential parameters, which is pivotal for CR [31]. Contrary to that, Martin et al. demonstrated no significant correlation between OSCE scores, data interpretation and CR [43]. These factors raise the question as to whether aviation-like SA training and assessment could be purposefully reflected in medical education and assessment. OSCEs may be a suitable instrument to teach and evaluate students’ use of SA as part of their clinical reasoning. The purpose of this paper is to review the literature with a view to identifying whether levels of SA can be assessed during undergraduate medical training utilising OSCEs based on Endsley’s model.

## Methods

**Fig. 1:**
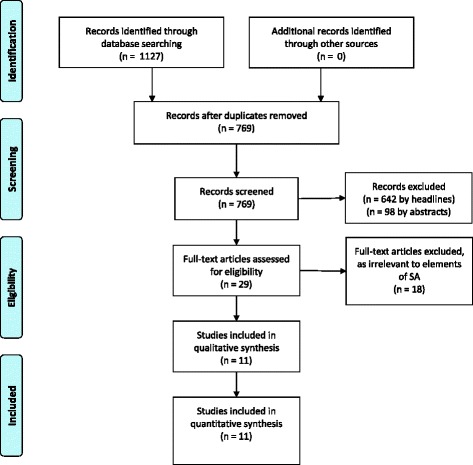
PRISMA Flowchart

A systematic search of the literature was performed pertaining to SA and OSCEs, to identify studies published between January 1975 (first paper describing an OSCE) and February 2017, in peer reviewed international journals published in English**.** PUBMED, EMBASE, PsycINFO Ovid and SCOPUS were searched for papers that described the assessment of CR using OSCEs among undergraduate medical students. Key search terms included “Objective Structured Clinical Examination”, “Objective Structured Clinical Assessment” or “OSCE” and “non-technical skills”, “sense-making”, “clinical reasoning”, “perception”, “comprehension”, “projection”, “situation awareness”, “situational awareness” and “situation assessment”. Boolean operators (AND, OR) were used as conjunctions to narrow the search strategy, resulting in the limitation of papers relevant to the research interest (Table 1). Publications relating to undergraduate medical training and ‘situational awareness’ or information processing as part of clinical reasoning were included. Due to different cognitive demands and scopes of practice, publications relating to nursing, paramedical disciplines, pharmacy and veterinary education were excluded from the search. The abstracts of remaining papers were manually reviewed in order to ensure their relevance. Areas of particular interest were elements of SA within OSCEs and the assessment of SA within these examinations. Additionally, a manual review of the references listed in the remaining publications was carried out and any publications of potential interested were sourced and reviewed (selection process described in Fig. 1).

**Table 1:** Steps of initial literature search to retrieve papers for the critical appraisal for their relevance to SA and OSCE in undergraduate medical education

1	Objective structured clinical examination
2	OSCE
3	OR 1–2
4	Objective structured clinical assessment
5	OR 3–4
6	Non-technical skills
7	AND 5–6
8	Sense-making
9	AND 5–8
10	Clinical reasoning
11	AND 5–10
12	Perception
13	Comprehension
14	Projection
15	OR 12–13-14
16	AND 5–15
17	Situation awareness
18	Situational awareness
19	Situation assessment
20	OR 17–18-19
21	AND 5–20

## Results

The search of the literature retrieved 11 articles eligible for inclusion (Table 2). Only one publication demonstrated an association between the OSCE and SA. An appraisal of the study design of the utilised simulation scenario, however, revealed that a root cause analysis was undertaken by the medical students to identify a prescription error [44]. Part of the examination focused on SA Level 1 when students were asked to take a history of the incident and SA Level 2 when integrating this data into the understanding of the situation. The authors suggested OSCEs to reflect utilisation of SA, however, neither a definition of the meaning nor the model of SA used for the conclusion was provided. Evaluation of SA Level 1 were identified in 11 publications, mostly seen in elements such as physical examinations, history taking but also in obtaining an overall impression of the patient and the retrieval of diagnostic test results. All 11 studies demonstrated continuative evaluation of elements of SA Level 2, demonstrated by the integration of the gathered parameters in SA Level 1 into further information processing steps. Only two studies assessed the selection process of optional diagnostic and treatment modalities categorised in SA Level 3.

**Table 2:** Results of the analysis of 11 identified papers concerning SA (SA Level 1,2,3 Column 5,6,7, respectively) in undergraduate medical training evaluated by OSCEs

Author / year of publication	Year of study	Number of students	Level of education	SA Level 1	SA Level 2	SA Level 3	Feedback	Assessment tool for SA	Educational tool for SA	Research interest
Volkan 2004 [49]	1999	169	year three	History taking, physical examination	Differential diagnosis	Consideration of treatment options		X		Factor analysis of OSCE constructs
Durak 2007 [51]	2000–2001	382	year six	Overall impression, history taking, diagnostic test results	Differential diagnosis	Consideration of treatment options, identification the need for further investigations	X		X	Case-based stationary examination
Varkey 2007 [44]	2003	42	year three	History taking	Identification of root cause of error		X		X	Root-cause analysis of error
Durning 2012 [45]	2010	170	year two	History taking, physical examination	Differential diagnosis			X		Feasibility, reliability, and validity of the evaluation of clinical reasoning utilising OSCEs
Myung 2013 [53]	2011	145	year four	Physical examination	Differential diagnosis				X	OSCE evaluation impact of pre-encounter analytical reasoning training
Lafleur 2015 [52]	2013	40	year five	Physical examination	Diagnostic reasoning				X	Influence of OSCE design on diagnostic reasoning
LaRochelle 2015 [46]	2009–2011	514	year four	History taking, physical examination	Clinical reasoning			X		Impact of pre-clerkship clinical reasoning training
Park 2015 [47]	2011	65	year four	Overall impression, history taking, physical examination	Differential diagnosis			X		Comparison of clinical reasoning scores and diagnostic accuracy
Sim 2015 [48]	2013	185	year five	History taking, physical examination	Data interpretation, clinical reasoning			X		Assessment of different clinical skills using OSCE
Stansfield 2016 [50]	2012	45	year four	Physical examination	Diagnostic reasoning			X		Evaluation of embedding clinical examination results into diagnostic reasoning
Furmedge 2016 [54]	2013/ 2014	1280	year one/ two	Information gathering	Predefined focus on integration of basic and clinical science		X		X	Acceptability and educational impact of OSCEs in early years

**Fig. 2:**
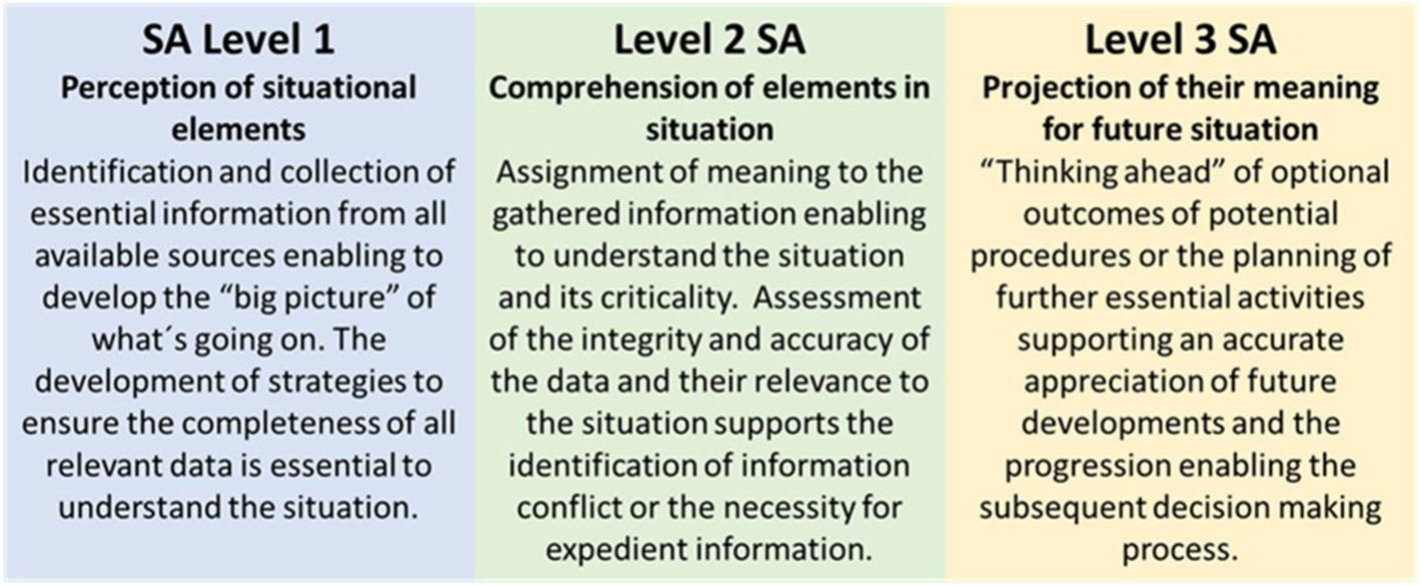
Levels of SA based on Endsley’s model [17]

Six papers described the OSCE as having the potential to be an assessment tool for CR [45–50], a method that might correspond with those used for the assessment of SA in high-risk environments or simulation scenarios (as described in Fig. 2). Furthermore, five papers suggested the OSCE as a valuable means for educating medical students on information gathering and processing when they are assessing the identification of the clinical presentation and incorporating the findings into their decision tree [44, 51–54].

### Situational awareness as part of the evaluation of clinical reasoning

Six studies concluded that OSCE stations allow for the assessment of students’ utilisation of CR abilities within diagnostic thinking [45–50]. In a study by Durning et al. based on three successive stations, students were asked to take a history from a patient, synthesise the data and provide the most likely diagnosis and a problem list. In the last step, the patient had to be presented to an attending colleague [45]. La Rochelle and colleagues detected a correlation between clinical and reasoning skills during pre-clerkship and abilities observed during internship [46]. Therefore, they suggested the potential of OSCEs to identify and foster those students who are experiencing difficulties with diagnostic reasoning and so possibly to prevent problems in subsequent clinical performance. Park et al., in contrast, demonstrated the inability of OSCE scores to correlate with CR abilities [47]. However, they demonstrated that scores achieved in CR OSCEs strongly correlated with diagnostic accuracy. When assessing students across 16 OSCE stations, Sim et al. demonstrated, that out of six evaluation criteria [history taking, physical examination, communication skills, CR skills, procedural skills, professionalism] procedural skills were identified as strongest and CR abilities as weakest [48]. They suggested that the low mean scores could be the result of students` lack of biomedical knowledge, their inability to incorporate the collected information into the clinical presentation of the patient or a combination of both. Volkan et al. in their study suggested two fundamental structures for OSCEs. Information gathering was represented by history-taking and physical examination, whereas reasoning and dissemination included hypothetico-deductive testing and differential diagnostic thinking [49]. Based on the findings of previous studies in which students showed a drop in CR when focussing on history-taking and physical examination, they highlighted the importance of comprehensive OSCEs to assess the ability to apply both processes simultaneously. In an innovative OSCE assessing the connotation of CR and physical examination abilities, Stansfield and colleagues identified a discrepancy between integrating acquired knowledge into the selected physical manoeuvres [50]. Additionally, there were fewer deficits in employing adequate physical examination skills in students able to embed their findings into the CR process.

### The OSCE as an educational tool for situational awareness

Five research groups identified the potential for OSCE stations to be teaching tools for SA within medical education [44, 51–53]. Generally, studies demonstrated better diagnostic accuracy and reasoning abilities among students when using an underlying analytical approach. Direct feedback or the addition of supportive information between incremental OSCE scenarios exemplified good educational properties. Durak et al. described a model in which hybrid forms of OSCE stations were applied [51]. Based on patient scenarios, students were asked to develop a treatment plan and were guided in a stepwise manner. The initial step included the collection of relevant data from history-taking, evaluating signs and symptoms, and the identification of underlying pathophysiological changes. After identifying the most likely diagnosis, students were probed to extract relevant information from the clinical notes and diagnostic results. Subsequently, students created the treatment plan for the patient based on the chosen diagnosis. In between these steps, corrective feedback was provided and incorporated into subsequent decision making. This method was found to be a motivator for students to improve their CR. Lafleur et al. observed the impact of the design of OSCE stations on the learning behaviour of students [52]. They described students applying more diagnostic reasoning when studying for whole task OSCEs rather than those that focused purely on physical examinations. Backward and forward associations, that is, either looking for evidence to support a suspected diagnosis or the aggregation of all identified symptoms and signs to conclude a diagnosis respectively, are both tasks that demand higher cognitive processing activities and, were strengthened when studying collaboratively for comprehensive OSCEs. Myung et al. compared analytical reasoning ability and diagnostic accuracy in a randomised controlled study [53]. On analysis of two groups of students, one of which had received prior education on analytical reasoning and one of which had not, OSCE scores achieved in both cohorts demonstrated no difference for information gathering. However, higher diagnostic accuracy was seen in that group of students which had received training in applying analytical reasoning strategies. Due to the similarity to real clinical situations, Varkey et al. suggest that OSCEs in general are an ideal tool for assessing and teaching SA [44]. However, no statement of the meaning of SA or the association with the healthcare environment was provided. In their study, students were asked to identify pivotal information in an error-induced patient encounter. Formative feedback was provided by the tutor on information gathering, root cause analysis, and completing the task. Furmedge and colleagues interrogated the appreciation of students for a novel, formative OSCE. The clinical scenario was designed to enable testees to exemplify the integration of skills and knowledge into the understanding of a situation rather than the pure retrieval of recited text passages. In this study, OSCEs were seen as a learning environment to develop cognitive strategies when exposed to clinical scenarios mirroring reality [54].

## Discussion

**Fig. 3:**
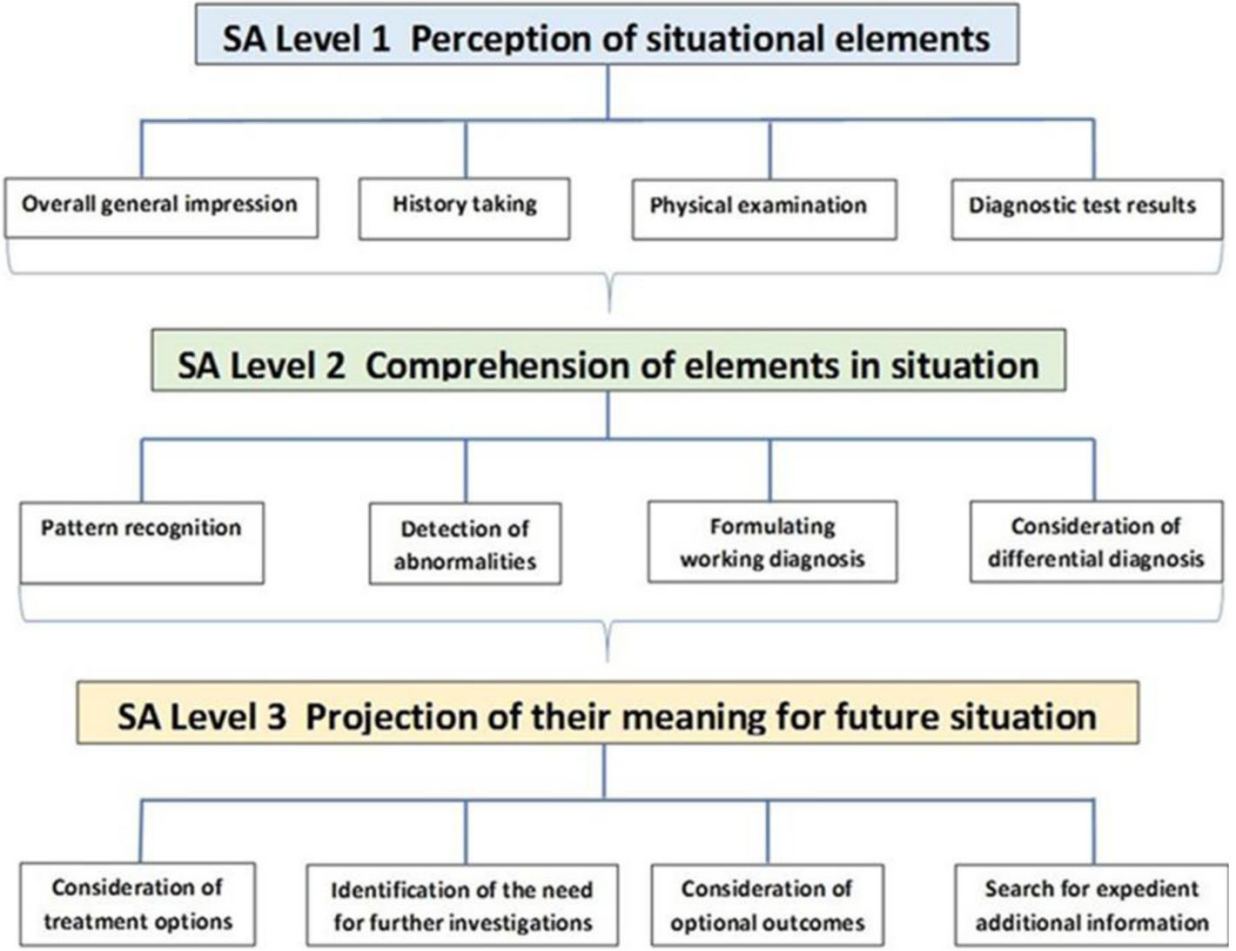
Elements of SA in the clinical context

**Fig. 4:**
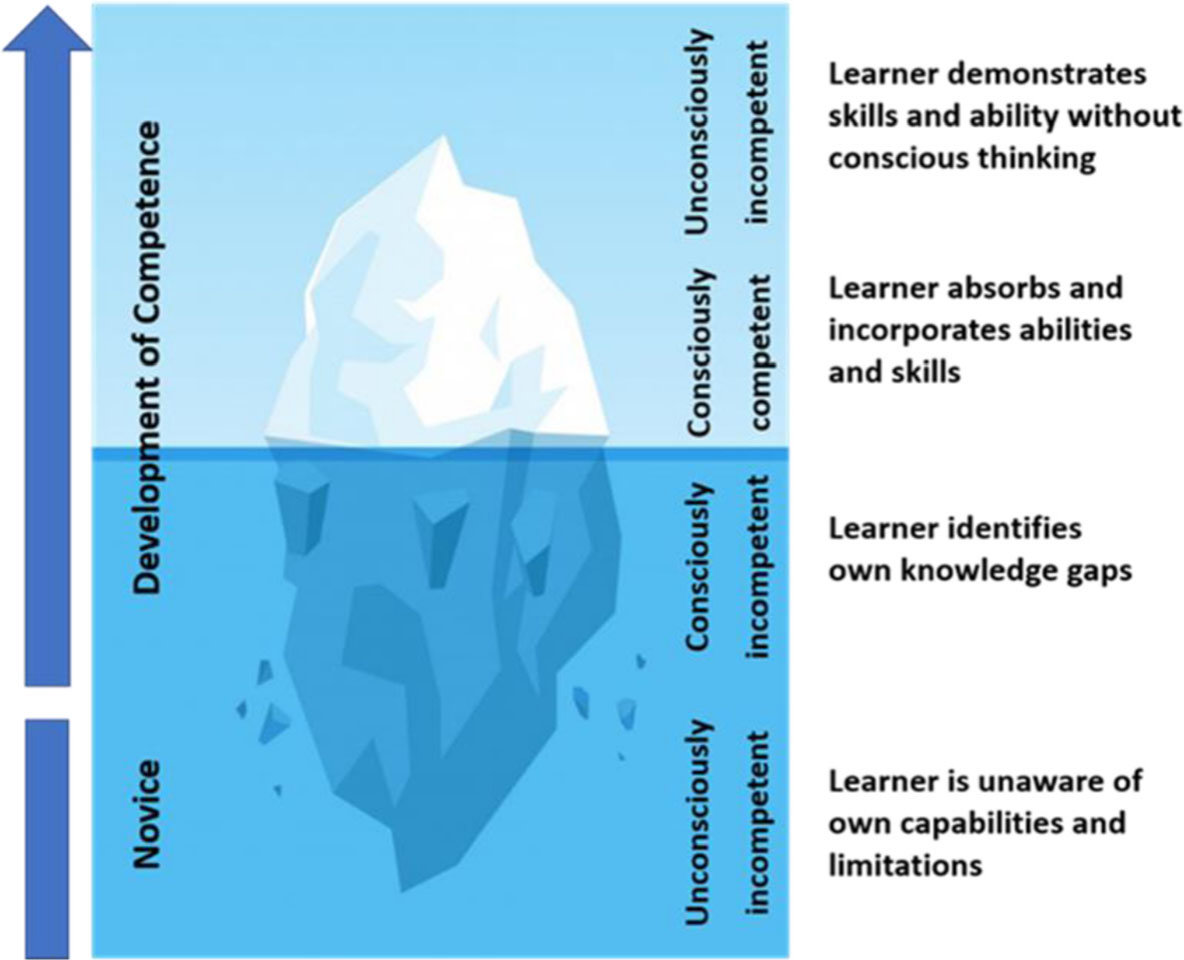
Developmental stages in compentence according to Scott [69] (designed by Vv studio Freepik.com)

We suggest that OSCE stations could be utilised for the assessment of elements of SA (Fig. 3) in medical students, using whole task simulation scenarios. So far, no distinct comprehensible methodology has been described which is universally accepted as fundamental measurement of SA. Furthermore, the conjecture that accurate SA automatically correlates with adequate performance and vice versa has been disproven. Although students may demonstrate history-taking, physical examination and procedural skills, the literature suggests that they are frequently unable to embed their findings in subsequent steps and decisions. This might be explained by the fact that novices often only recite enormous amounts of information from their “knowledge database”. Reduced diagnostic accuracy by medical students accentuated the primary necessity for efficient data gathering and processing [29, 38]. Diagnostic excellence has been suggested to originate from a reasonable understanding of the fundamental anatomical and physiological context in conjunction with pathophysiological changes potentially identifiable within elements of SA in any given clinical presentation [55]. Borleffs et al. described the objective of teaching CR as the ability to make correct decisions in the process of establishing a diagnosis [56]. Alexander concluded that students must be able to demonstrate how to do it, but also, at the same time, why to do it [57]. Zwaan et al. suggested implementing interventions with proven records to enhance SA within the diagnostic reasoning process [8]. Gruppen and colleagues depicted how the different utilisation of hypotheses and information depends on clinical experience and expertise [58]. In their study, the collection and appropriate selection of data was demonstrated to be more difficult than the pure integration of available information. This imbalance between efficient information gathering and successive data integration suggests that educational measures should aim to enhance procedures in collecting and processing relevant information (Fig. 4).

### The OSCE as a learning approach for SA for medical students

OSCE stations can be educational tools for CR, pattern recognition and problem-based learning [59]. To foster the ability of putting it all together, Furmedge et al. suggested an early exposure of students to OSCEs [54]. However, they concurrently highlighted the need to identify how early OSCE exposure could contribute to development of non-analytical reasoning skills. When analysing feedback upon completion of the OSCE cycle, Haider and colleagues summarised students` appreciation of this type of assessment, which supported their individual abilities to identify areas of clinical weakness, thus inspiring their interest in developing information processing skills [60]. Baker et al. introduced three strategies for developing CR, hypothesis testing, forward thinking and pattern recognition [61]. They developed a specific assessment tool for the interpretative summary, differential diagnosis, explanation of reasoning and alternative diagnostics [IDEA]. OSCEs were described as a means of valuable feedback for both, examinee and educator [62], that enables the reinforcement of the importance of SA as an underlying requirement for well-informed CR in all disciplines [19, 29]. Feedback provided upon completion of OSCE scenarios could support the faculty’s appraisal and the examinees` self-rating of the sense-making process when selecting best clinical diagnosis and therapeutic options [51]. Providing individualised feedback upon completion of the OSCE was described as being complex [63]. Thus, establishing the cognitive map of the underlying information processing could potentially identify why selected parameters and criteria during the CR process either made sense to the testee at the time or were neglected [64–66]. Remedial teaching and education at undergraduate level could be considered if a deficiency within the three levels of SA was identified during OSCE assessments [67]. Gregory et al. described an innovative method of teaching aspects of situational awareness in undergraduate medical training by exposing students not only to perils, but also to additional indications of a patient’s condition [68]. Upon entry into undergraduate training, students are exposed to a clinical area without a patient, such as the bed space, and are evaluated collectively in their ability to recognise any hazards and clues indicating supportive information about the clinical status of the patient. Students are also expected to extract additional parameters from clinical notes and diagnostic results. The positive feedback from students and tutors suggests that this approach is a promising tool in teaching SA to medical students.

## Conclusion

Assessment of elements of SA as adapted from the model by Endsley might have the potential to be translated into certain aspects of CR evaluation using OSCEs. Given that assessment is a fundamental driver of adult learning, incorporating the quantitation of utilisation of SA within OSCEs during undergraduate medical training could develop and strengthen teaching on information gathering and efficient processing. However, further research needs to establish whether different levels of SA can be identified throughout the medical curriculum and its assessment including the use of paper cases and reviewing medical records. If so, are these levels of assessment congruent with the learning outcomes in preclinical and clinical years? In order to teach students how to perceive and incorporate relevant data, it is essential to provide focussed and informative feedback related to each level of SA and the associated steps of CR. Upon identification of the potential and ability to assess levels of SA in a curriculum e. g. OSCEs, we suggest that students be exposed, in a staged format, to the concept of SA at the early stages in their training, prior to meeting complex challenging clinical situations in their later medical careers. Efforts in conveying underlying elements of SA during undergraduate education could be reflected in enhanced abilities to read and understand clinical scenarios in subsequent clinical practice.

## Abbreviations

CR: Clinical Reasoning
OSCE: Objective Structured Clinical Examination
SA: Situational Awareness
